# Profound human/mouse differences in alpha-dystrobrevin isoforms: a novel syntrophin-binding site and promoter missing in mouse and rat

**DOI:** 10.1186/1741-7007-7-85

**Published:** 2009-12-04

**Authors:** Sabrina V Böhm, Panayiotis Constantinou, Sipin Tan, Hong Jin, Roland G Roberts

**Affiliations:** 1 Division of Medical & Molecular Genetics, King's College London, London, UK

## Abstract

**Background:**

The dystrophin glycoprotein complex is disrupted in Duchenne muscular dystrophy and many other neuromuscular diseases. The principal heterodimeric partner of dystrophin at the heart of the dystrophin glycoprotein complex in the main clinically affected tissues (skeletal muscle, heart and brain) is its distant relative, α-dystrobrevin. The α-dystrobrevin gene is subject to complex transcriptional and post-transcriptional regulation, generating a substantial range of isoforms by alternative promoter use, alternative polyadenylation and alternative splicing. The choice of isoform is understood, amongst other things, to determine the stoichiometry of syntrophins (and their ligands) in the dystrophin glycoprotein complex.

**Results:**

We show here that, contrary to the literature, most α-dystrobrevin genes, including that of humans, encode three distinct syntrophin-binding sites, rather than two, resulting in a greatly enhanced isoform repertoire. We compare in detail the quantitative tissue-specific expression pattern of human and mouse α-dystrobrevin isoforms, and show that two major gene features (the novel syntrophin-binding site-encoding exon and the internal promoter and first exon of brain-specific isoforms α-dystrobrevin-4 and -5) are present in most mammals but specifically ablated in mouse and rat.

**Conclusion:**

Lineage-specific mutations in the murids mean that the mouse brain has fewer than half of the α-dystrobrevin isoforms found in the human brain. Our finding that there are likely to be fundamental functional differences between the α-dystrobrevins (and therefore the dystrophin glycoprotein complexes) of mice and humans raises questions about the current use of the mouse as the principal model animal for studying Duchenne muscular dystrophy and other related disorders, especially the neurological aspects thereof.

## Background

The dystrophins and dystrobrevins constitute two branches of a superfamily of membrane-associated cytoskeletal proteins found throughout the animal kingdom [1]. While invertebrates have one member from each class, the increased genomic complexity of vertebrates has conferred on them three paralogous members, namely dystrophin [2], utrophin [3] and dystrophin-related protein 2 (DRP2) [4] in the dystrophin branch and α-, β- and γ-dystrobrevin [1,5-9] in the dystrobrevin branch (γ-dystrobrevin has been lost in tetrapods). Dystrophins and dystrobrevins can heterodimerise to form the core of a widely expressed membrane-bound complex, the dystrophin glycoprotein complex (DGC). Mutations which disrupt the DGC cause numerous types of skeletal myopathy (including Duchenne muscular dystrophy, DMD) in humans and mice [10,11]. The DGC is often considered to function as a structural scaffold linking the extracellular matrix to the intracellular cytoplasm, thereby helping to resist the large mechanical forces experienced by contractile tissues. As well as resulting in progressive skeletal and cardiac myopathy, however, DMD mutations are also associated with substantial cognitive effects [12,13], suggesting an important and poorly understood role for DGCs in non-contractile tissues.

The heterodimerisation of dystrophins and dystrobrevins is mediated by their C-terminal coiled-coil domains [14,15], and it is generally assumed that all DGCs have a dystrophin/dystrobrevin core (indeed, in the nematode, for example, the null dys-1 and dyb-1 mutant phenotypes are indistinguishable [16]). The vertebrate dystrobrevins are expressed in a dynamic and complex way during development [17-19], but disruption of the α-dystrobrevin gene in mouse results in a very mild skeletal and cardiac myopathy with retention of the remainder of the DGC (apart from neuronal nitric oxide synthase, nNOS) and no evident central nervous system (CNS) defects [20]. This mouse model exhibits structural abnormalities of the neuromuscular junction [21] (NMJ) reminiscent of those caused by utrophin loss [22]. Disruption of the paralogous β-dystrobrevin gene gives no noticeable phenotype, but simultaneous ablation of both dystrobrevin genes results in appreciable reduction of GABA_A_-containing synapses on cerebellar Purkinje cells and a concomitant motor defect [23]. This last study showed conclusively that both dystrobrevins contribute to functionally important DGCs in CNS GABAergic synapses [23] in a manner similar to that of α-dystrobrevin in the NMJ, a cholinergic synapse [21].

One of the functions of dystrophins and dystrobrevins in the DGC seems to be to act as a platform for the recruitment of members of the syntrophin family. The syntrophins are multifunctional adaptor proteins which contain a postsynaptic density protein 95/discs large/zonula occludens-1 (PDZ) domain, two pleckstrin homology (PH) domains, and a C-terminal syntrophin-unique (SU) region. Numerous candidate ligands for the syntrophins have been identified, including nNOS [24], voltage-gated sodium channels [25], syntrophin-associated serine/threonine kinase (SAST) [26], diacylglycerol kinase-ζ [27], the lipid transporter ABCA1 [28], and aquaporin-4 [29], all of which interact with the syntrophins' PDZ domain. The first PH domain is proposed to be involved in an interaction with lipid headgroups at the membrane [30,31]. The second PH domain and the SU domain mediate interaction with the dystrophins and dystrobrevins [32]. There are five vertebrate syntrophins (α1-, β1-, β2-, γ1-and γ2-syntrophins) [33-35], arising from two invertebrate common orthologues, syntrophin-1 (α/β-like) and syntrophin-2 (γ-like) [36].

The syntrophin-binding sites (SBSs) on dystrophins and dystrobrevins have been identified [37]. They appear to be short, modular and largely α-helical motifs, of which there are two (SBS1 and SBS2) in most dystrophin family members (vertebrate or invertebrate). Invertebrate dystrobrevins have a single SBS, but in vertebrates a tandem duplication of the SBS-encoding exon has resulted in the potential for two SBSs (again, SBS1 and SBS2) in all dystrobrevins except fish β-dystrobrevins [1]. It is striking that the SBS-encoding exons of dystrophin and α- and β-dystrobrevin [37,38] are subject to alternative splicing, meaning that syntrophin stoichiometry can be regulated, in principle at least, from two to four syntrophin molecules per DGC [37]. There is a suggestion from at least one study, however, that not all SBSs are equal, and that there is a degree of SBS-syntrophin specificity [39].

Almost all of our knowledge regarding the function of α-dystrobrevin has been gleaned from the mouse; surprisingly, apart from one isolated report of a dominantly inherited heart phenotype [40], no human disease has yet been associated with mutations in the α-dystrobrevin gene. We report here the unexpected finding that most tetrapod α-dystrobrevin genes (including human, but not mouse or rat) have the potential to encode a third functional SBS, SBS1'. Furthermore, we show explicitly that the N-terminally truncated α-dystrobrevin isoforms 4 and 5, first described in human tissues [8] and rarely studied since, are specifically ablated in the murids. We use this as a starting point to compare the tissue-specific diversity of human and mouse α-dystrobrevin transcripts, and show that more than half of the usual mammalian α-dystrobrevin brain isoforms are non-existent in mouse.

## Methods

### Samples

Human tissue RNAs were purchased from Ambion (Austin, Texas, USA). Mouse RNA was extracted from tissues (a gift from Dr Rebecca Oakey, King's College London, UK) obtained from normal adult C57BL6-J mice killed by cervical dislocation. A QIAGEN RNA extraction kit was used according to the manufacturers' instructions (QIAGEN, Hilden, North Rhine-Westphalia, Germany). The lamprey RNA was a kind gift from Dr Sebastian Shimeld (University of Oxford, UK). RNA was quantified using an RNA Nano Chip on an Agilent 2100 Bioanalyser (Agilent Technologies, Santa Clara, California, USA).

### Non- and semi-quantitative RT-PCR

Single-strand cDNA was synthesized from equal amounts of total RNA using gene-specific primers (Additional file 1, Table S1 and S3) and M-MLV Reverse Transcriptase (Applied Biosystems, Foster City, California, USA); random hexamer primers were used for synthesis of cDNA for endogenous controls. 2 βl cDNA was used for the first round of nested polymerase chain reaction (PCR) using *coarse *isoform-defining primer pairs (Additional file 1, Tables S1 and S3). The first-round product was diluted five-fold and was used as template for the second-round PCR using *fine *isoform-defining primer pairs (Additional file 1, Tables S2 and S4). All primer sequences and PCR conditions are given in Additional File 1. A degree of quantitation was achieved by stopping the second-round PCR at 5-cycle intervals and electrophoresing the products on ethidium bromide-stained agarose gels.

### Quantitative RT-PCR

Human and mouse second-round nested reverse transcript-PCR (RT-PCR) products were cloned into pCR4-TOPO (Invitrogen, Carlsbad, California, USA) and verified by sequencing. Plasmid preparations containing the appropriate inserts were linearised using *Nco*I or *Pme*I (New England Biolabs, Ipswich, Massachusetts, USA), quantitated using a Nanodrop ND-1000 spectrophotometer (Labtech International, Ringmer, East Sussex, UK), and serially diluted. These were used as standards to allow comparison between different amplimers. *Coarse *isoform-defined first-round RT-PCR products were used as templates in a quantitative *fine *isoform-specific second round PCR (see Additional File 1 for conditions) using SYBR green detection in an ABI Prism 7000^® ^Sequence Detection System (ABI, Foster City, California, USA). For each amplimer, measurements of threshold cycle (Ct) were used to infer initial template copy number (in the common first-round PCR) when compared to Ct measurements of the respective serially diluted standard. Each datum plotted is the mean of three measurements, normalised to 18S rRNA copy number to control for RNA loading.

### Yeast two-hybrid assay

The human α-dystrobrevin bait constructs comprise segments of human α-dystrobrevin cDNA inserted into the *Nco*I/*Bam*HI sites of vector pGBKT7 (Clontech, Mountain View, California, USA). These consist of the last 12 codons of exon 10, then either exon 11b (isoform b), exons 12 and 13 (isoform c), or neither (isoform a), followed by exon 14 and the first seven codons of exon 15 (see Additional file 2 for details). These wild-type constructs were then mutated at one or both of the SBSs, using a mutagenic PCR primer to introduce a leucine-to-proline mutation. Wild-type and mutant pGBKT7-ADyb constructs were co-transformed into AH109 host yeast cells with either empty pGAD10 prey vector (as a negative control) or a pGAD10 construct containing the entire rat β1-syntrophin coding sequence (as the experiment). Co-transformed yeast was plated onto minimal media plates lacking either leucine and tryptophan (LT-, as a control for transformation efficiency) or adenine, histidine, leucine and tryptophan (AHLT-, as a test for interaction). Only yeast containing bait and prey constructs which encode interacting proteins should grow on AHLT- plates. Co-transformation of pGBKT7-p53 and pGAD7-T-antigen (Clontech) was used as a positive control.

### New GenBank accession numbers

[GenBank: FJ535564], [GenBank: FJ535565], [GenBank: FJ535566], [GenBank: FJ535567]

## Results

**Figure 1:**
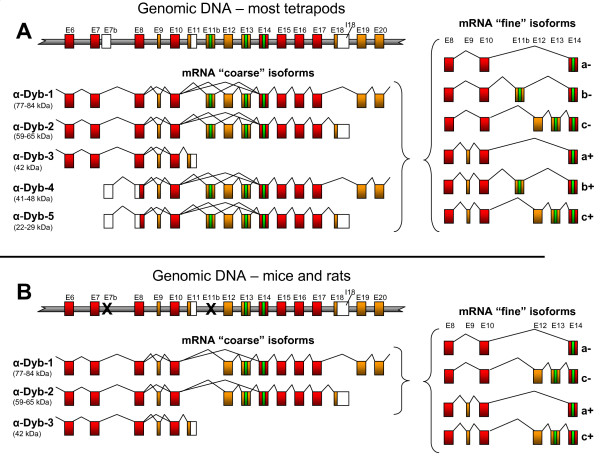
**Comparison of α-dystrobrevin gene structure and isoforms in humans and mice**. The central genomic DNA structure and observed transcript isoforms are shown for **A**) most tetrapods, including humans, and **B**) mice and rats. In each instance, the partial genomic DNA structure (not to scale) is shown at the top, with the *coarse *isoforms (numbered as in Peters *et al*. [42]) shown below. To the right are shown the *fine *isoforms generated by alternative splicing of the SBS-encoding region, labelled from 'a' to 'c' according to the SBS content, and '+' or '-' according to whether tiny exon 9 is included. We suggest an extension to the existing nomenclature system, such that, for example, a transcript starting with exon 7b and ending at the polyadenylation site in intron 18, containing exon 11b spliced to exon 14 and including exon 9, would be called 'α-dystrobrevin-5b+'. Red boxes - constitutive coding exons. Orange boxes - alternatively spliced coding regions. White boxes - untranslated regions. Green boxes - encoded SBSs. Black crosses - exons disrupted in murids. Exon numbering according to Ambrose *et al*. [41], apart from exons 7b and 11b, which are described first here.

The vertebrate α-dystrobrevin gene has a complex gene structure, with multiple promoters, alternative 3' exons and alternatively spliced exons. Throughout this paper, we will use the exon nomenclature described by Ambrose *et al*. [41] and isoform nomenclature from Peters *et al*. [42]. The particularly complex central region of the gene is represented in Figures 1A and 1B - features include an alternative first exon in intron 7 (which initiates the N-terminally truncated isoforms α-dystrobrevin-4 and -5) [8], alternative splicing of exons 9, 12 and 13 [6,8], and an alternative last exon (exon 11) whose use results in the C-terminally truncated α-dystrobrevin isoform (α-dystrobrevin-3) [8]. Exons 13 and 14 encode SBS1 and SBS2, respectively, with exon 13 having arisen through duplication and divergence of the ancestral invertebrate exon 14. We distinguish between *coarse *isoform variants (those which differ at a gross structural level through the use of alternative promoters or alternative last exons; indicated by numerical isoforms 1-5) and *fine *isoform variants (those which differ subtly through alternative internal exon usage) - see Figure 1, right hand side.

### Discovery of a novel α-dystrobrevin coding exon

**Figure 2:**
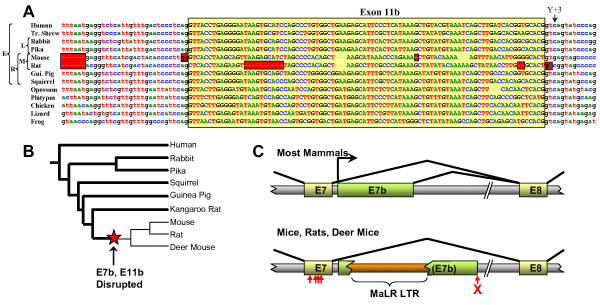
**Exon 11b and exon 7b are highly conserved but disrupted in murids**. **A**) The murid exon 11b sequence has experienced multiple catastrophic defects, including frameshifting deletions and mutation of acceptor (mouse), donor (rat) and lariat branch (both species) sites. Red boxes - probable deleterious mutations. Yellow box - exon 11b. Arrow - unusual pyrimidine at +3 position. Broad taxonomy is shown at left; E - superorder Euarchontoglires; L - order Lagomorphia; R - order Rodentia; M - family Muridae. **B**) Selected phylogeny of superorder Euarchontoglires (based on Huchon *et al*. [61]), showing species in which exon 7b and exon 11b are known to be intact (bold lines) or disrupted (faint lines). Red star indicates most parsimonious time of mutation of both elements. **C**) Disruption of murid exon 7b by retroviral insertion. The upper panel shows the exon structure present in most mammals (to scale), while the lower shows that found in murids. Large horizontal arrow - majority transcriptional start site observed in non-murid transcript sequences. Red cross - donor splice site mutation. Small vertical arrows - murid-specific substitutions in exon 7. A detailed annotated alignment of this mammalian genomic region appears in Additional file 3.

We were studying the genomic sequence of α-dystrobrevin genes in order to assess the conservation of exon 11, with a view to identifying 3' untranslated regions for a zebrafish expression study [17]. During this process, we noted that most amniote α-dystrobrevin genes contained, adjacent to exon 11, an unannotated patch of sequence which was strongly conserved from human to chicken. Closer examination revealed conserved consensus splice sites and a strong resemblance of the encoded peptide to those encoded by exons 13 and 14. We call this exon *exon 11b *and its encoded SBS *SBS1 *(Figures 1A and 2A). The immediate implication of this is that the α-dystrobrevin gene has the potential to encode not two, but three SBSs.

### Sequence characteristics, conservation and destruction of exon 11b

Exon 11b is observed in genomic sequences between exons 10 and 12 of α-dystrobrevin genes from almost all sequenced amniote genomes. Thus it is seen in most mammals (including opossum and platypus), and in chicken and anole lizard (Figure 2A). Although the genomic α-dystrobrevin sequence of the amphibian *Xenopus tropicalis* does not appear in the current build of the genome, there is a candidate exon 11b sequence in the NCBI trace archive. Exon 11b could not be found in teleost (bony) fish α-dystrobrevin gene sequences, but teleosts have notoriously derived genomes and may have lost exon 11b secondarily. No complete genomic sequence for cartilaginous fish α-dystrobrevin is available, and although the single lamprey dystrobrevin gene has a copy of exon 13, there is a gap in the *Petromyzon marinus* genome sequence between exons 10 and 12. Thus exon 11b is likely to be present in most amniotes, but may have originated earlier (and may be present in amphibia and/or cartilaginous fish). No obvious equivalent of exon 11b is seen in vertebrate β-dystrobrevin or fish γ-dystrobrevin genes.

The only mammalian genomes found to lack an intact exon 11b sequence were the mouse (*Mus musculus*) and rat (*Rattus norvegicus*), in which splice sites were mutated (acceptor AG→GG in the mouse, donor GT→GA in the rat, and lariat branch site deleted in both species) and multiple independent reading frame shifts (two in rat, one in mouse) have occurred (Figure 2A). This, combined with the fact that expression of α-dystrobrevin has been most intensively studied in the mouse [43], probably accounts for the historical failure to recognise exon 11b. By contrast, the exon's reading frame and splice sites are intact in other rodents (the guinea pig *Cavia porcellus*, the squirrel *Spermophilus tridecemlineatus *and the kangaroo rat *Dipodomys ordii*). As rats and mice form part of a monophyletic group (family Muridae, subfamily Murinae) within the order Rodentia, it seems that exon 11b function was specifically lost in a common ancestor of these two species (Figure 2B).

Previous reports and GenBank entries have described two main isoforms of α-dystrobrevin transcript in the SBS-encoding region, namely those with one SBS (SBS2) in which exon 10 is spliced directly onto exon 14, and those with two SBSs (SBS1 and SBS2) which include exons 12 and 13 between exons 10 and 14. A BLASTn search for human expressed sequence tags (ESTs) (in the EST database, dbEST, Sept 2007 release) using an artificial composite transcript sequence (exons 8-9-10-11b-12-13-14-15) revealed, surprisingly, many more sequences containing exon 11b than containing exon 12 and/or 13. Indeed, of those ESTs which traverse exons 10 and 14, 10 out of 45 (22%) contain exon 11b; most of the remainder splice exon 10 directly onto exon 14. Exons 12 and/or 13 are only seen in two ESTs. Thus exon 11b usage in human tissues seems to be significant.

Like the alternatively spliced exon 13 of all α- and β-dystrobrevin genes (and exon 78 of the dystrophin gene), but unlike the constitutively spliced exon 13 of all γ-dystrobrevin genes, all exon 11b sequences examined have the atypical pyrimidine at position +3 of the donor splice site (Figure 2A). We have previously speculated [1] that this sub-optimal donor splice site (base +3 is only a pyrimidine in <4% of vertebrate splice sites [44]) might render the cellular decision whether to include or exclude such exons more sensitive to the presence of particular SR proteins or other trans-acting factors [45].

### Intron 7 (α-dystrobrevin-4 and -5) transcripts

When the human α-dystrobrevin gene was first described over ten years ago [8], two N-terminally truncated isoforms, now known as α-dystrobrevin-4 and -5, were noted. These start from an isoform-specific first exon in canonical intron 7. Strangely, despite their frequent representation in α-dystrobrevin isoform diagrams, these isoforms have barely been studied since (their apparent absence from mice is noted in passing by Peters et al. [42]). We noted that a major difference between the human and mouse dbEST content (in addition to the absence of exon 11b in mouse) is the existence of large numbers of human ESTs which commence in intron 7 (i.e. isoforms α-dystrobrevin-4 and -5); in contrast, no mouse ESTs start in intron 7. This constitutes the major difference between the EST profiles of the human and mouse genes, and most other differences arise from this skewing of the transcript population.

We decided to examine further the structure and prevalence of ESTs which start in intron 7. The vast majority (37 out of 50) of the human intron 7 ESTs start within a 30-bp window about 25 bp 3' of exon 7, suggesting a relatively tight transcriptional start site. Two of these are derived from cap-trapped libraries, indicating a genuine transcriptional start event rather than artifactual reverse transcriptase stalling. In addition, eight clustered human CAGE (cap analysis of gene expression) tags, which are also cap-trapped, lie in this 30-bp region [46]http://gerg01.gsc.riken.jp/cage/hg17prmtr/ (Additional file 3). All informative ESTs used the same donor splice site, 317 bp after the end of exon 7. This builds a picture of the first exon of the α-dystrobrevin-4 and -5 transcripts as a region of 270-290 bp lying 25 bp downstream of exon 7 (Figure 2C, and Additional file 3). All ESTs using this exon are derived from libraries prepared from the brain or parts thereof. We call this exon 'exon 7b'.

We found that dbEST (Oct 2007 release) contains, in addition to these human ESTs, three exon 7b-containing ESTs from chimpanzee (*Pan troglodytes*), one from the rhesus macaque (*Macaca mulatta*) and three from cattle (*Bos taurus*). All of these start very soon after exon 7 and then splice onto exon 8 using the same donor splice site as the human ESTs (Additional file 3). Like their human counterparts, they all derive from brain cDNA libraries. Given that there are similar proportions of exon 7b ESTs for human (34 out of ~8 million) and cattle (3 out of ~1.5 million) but not for mouse (0 out of ~4.8 million), it seems a safe assumption that transcription from this region is ancestral in eutherian mammals but disrupted in mice. A single opossum EST (EC290283) with the same start site but using a distinct donor splice site suggests an even earlier origin for this promoter. Chicken and frog have potential donor splice sites but there is no database evidence for their use.

Analysis of an alignment of the exon 7/exon 7b region from a range of mammalian species shows the exon 7b donor splice site to be conserved in most species (including platypus, a monotreme, and to a certain extent, chicken and lizard; Additional file 3). It is, however, absent from the opossum (which uses an upstream donor), and almost certainly compromised in mouse and rat (where an insertion of an A residue, GTAAGT→GTAAAGT, means that it lacks the G+5 present in 84% of mammalian splice sites[44]). The mouse and rat also share a ~360-460-bp insertion of a representative of the Mammalian Apparent LTR-Retrotransposon (MaLR) family [47] (Figure 2C and Additional file 3). As is the case for exon 11b, in the other available rodent genome sequences (*S. tridecemlineatus*, *C. porcellus*, and *D. ordii*) the MaLR insertion is absent and the donor splice site intact, suggesting that loss of exon 7b function is murid-specific (Figure 2B).

The site of the promoter itself is not obvious - we used MultiTF http://multitf.dcode.org/ to search for transcription factor binding sites (TFBSs) conserved between mammalian species known to express exon 7b transcripts. This revealed conserved TFBSs only within exon 7 itself. Indeed, although exon 7 is spectacularly highly conserved (97% identical between human and elephant), it has incurred four murid-specific subsitutions (vertical arrows in Figure 2C, cyan boxes in Additional file 3), reducing identity to only 92% between human and mouse. These disrupt three otherwise absolutely conserved TFBSs (for the transcription factors MSX1, MEIS1 and NFκB). The candidate promoter contains no TATA box or CpG island. We attempted amplification of mouse α-dystrobrevin-4 and -5 transcripts from a range of tissues using primers orthologous to those used in our human study (see below), but these yielded no products.

### Assessment of α-dystrobrevin transcript diversity in human tissues

We decided to use quantitative RT-PCR to assess the structure and representation of human α-dystrobrevin transcripts in four major sites of expression, namely skeletal muscle, heart, brain and colon. As a preliminary study we devised a semi-quantitative RT-PCR method which aimed to avoid several potential sources of bias (Additional file 1). Products were assessed for their approximate quantity and sequence to ascertain their structure and thereby broadly establish the transcript repertoire. These data showed that exon 10 is spliced faithfully onto exon 11b in a substantial proportion of brain transcripts, and that exon 11b is generally followed by either exon 14 (in the majority product [GenBank: FJ535565]) or very rarely by exon 12 (in the minority product [GenBank: FJ535564]). Exon 12 is almost always followed by exon 13, so that the three main *fine *isoforms observed are (in descending order of abundance) E10-E14, E10-E11b-E14, E10-E12-E13-E14. These isoforms contain one, two, and two SBSs, respectively. We suggest a refinement of the nomenclature to capture this complexity, namely that these three most prevalent *fine *isoforms should be given the suffix a, b and c, respectively (Figure 1, right). All three of these *fine *isoforms were seen either with (in the minority) or without (in the majority) the tiny 9-bp exon 9; we suggest that these be indicated by the additional suffix '+' and '-', respectively. Thus a transcript starting with exon 7b, ending in intron 18, containing exon 9 and splicing E10-E11b-E14 would be called 'α-dystrobrevin-5b+'.

**Figure 3:**
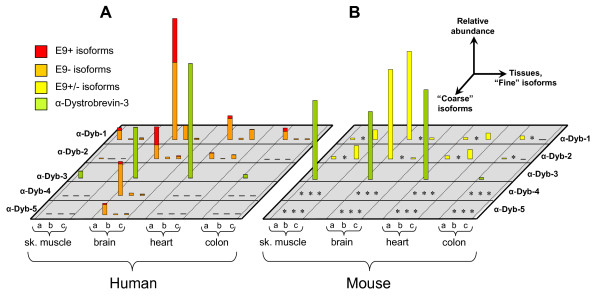
**Expression of α-dystrobrevin isoforms in human and mouse tissues**. The relative expression of the discernable *coarse *and *fine *isoforms in **A**) human and **B**) mouse heart, brain, skeletal muscle and colon, as determined by quantitative RT-PCR. As indicated, horizontal axes show the tissues tested and the *coarse *and *fine *isoforms, while the vertical axis shows the relative expression level in arbitrary units. For human tissues, presence and absence of exon 9 (+ and -) was also assessed; these are shown as differently coloured portions of the bars. Asterisks in mouse indicate isoforms which while present in other mammals, are non-existent in murids. For display purposes, brain α-dystrobrevin-1a was used to normalise between species. Although α-dystrobrevin-3 is not directly comparable to the other reactions, heart α-dystrobrevin-3 has been normalised to brain α-dystrobrevin-1a, an approximate equivalence that is evident in published northern blots.

Cloned products were used to generate dilution series as standards for quantitative nested RT-PCR. The outer reactions for this were deliberately chosen to be large, thereby avoiding size-dependent bias due to SBS splicing. In order to be able to ascribe SBS *fine *isoform splicing behaviour to their cognate *coarse *isoforms, we used outer reactions specific for the four relevant α-dystrobrevin *coarse *isoforms (Additional file 1, Tables S1 and S3) and inner reactions specific for the *fine *isoforms (Additional file 1, Tables S2 and S4). The relative quantities of all of these isoforms are presented in Figure 3A. As negligible amounts of E10-E12-E14 and the potential three-SBS 'd' isoform E10-E11b-E12-E13-E14 were observed, these are omitted.

Notable features of this isoform map include: a) expression of α-dystrobrevin-4 and -5 is entirely brain-specific (signal in brain is 3,000-12,000 times that in other tissues) and constitutes ~25% of the total brain α-dystrobrevin; b) the novel 'b' form, which contains exon 11b, makes up a fairly consistent 6% of isoforms in all tissues (9% in brain, where it is the second most abundant *fine *isoform); c) the canonical 'c' isoform seems to be largely heart-specific (33% of isoforms), with less in muscle and colon (14%, 7%) and very little in brain (3%); d) the ratios between α-dystrobrevin-1 and -2 and between α-dystrobrevin-4, -5 (i.e. the relative usage of the tyrosine-phosphorylated C-terminus) are relatively consistent between tissues, except for colon, where the C-terminus is always retained; e) the tiny 9-bp exon 9 is included in ~40% of brain α-dystrobrevin-1a and α-dystrobrevin-2a, but usually excluded elsewhere; f) α-dystrobrevin-3 expression is substantial in heart and brain.

### Comparison with mouse and other animals

We repeated this process, using orthologous primers where possible, in the cognate mouse tissues. The data are presented in Figure 3B. The principal differences from the human pattern are: a) absence of all 'b' isoforms as exon 11b is multiply disrupted (we did not attempt amplification of this isoform); b) absence of dystrobrevin-4 and -5 as exon 7b is not used (we attempted amplification of this isoform in all four tissues, but were unsuccessful); c) the 'c' isoform is the major fine isoform in skeletal muscle and heart (86%, 75%), as opposed to the 'a' isoform, which predominates in mouse brain and colon (98%, 97%) and in all human tissues tested; d) unlike humans, α-dystrobrevin-3 is highly expressed in mouse skeletal muscle.

**Figure 4:**
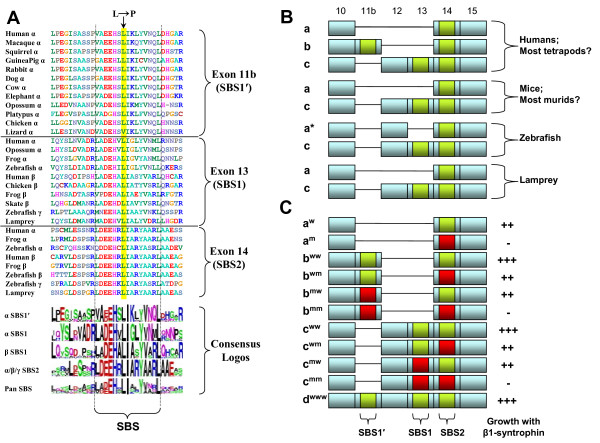
**The exon 11b encoded sequence is a functional SBS**. **A**) Comparison of exon 11b encoded protein sequence (containing SBS1') with those of other vertebrate SBS-encoding dystrobrevin exons. Vertical dotted lines delimit the 15-residue pan-SBS consensus, and specific SBS consensus sequence logos are presented below the alignment. Arrow at top indicates the leucine mutated to proline in the yeast two-hybrid constructs. **B**) Schematic diagram showing observed α-dystrobrevin SBS isoform repertoires in various vertebrates. 'a*' is a one-SBS isoform seen in zebrafish, where the 'a' form is not observed. **C**) Yeast two-hybrid analysis of the interactions between mutant and wildtype α-dystrobrevin constructs and full-length β_1_-syntrophin. *Fine *isoforms are shown as 'a', 'b', 'c', 'd'; mutant status of respective SBSs is shown as superscript 'w' (wild-type) or 'm' (mutant) and by green and red boxes, respectively. Growth on AHLT- plates was scored as '-' (0-10 colonies), '+' (10-100), '++' (100-1000) or '+++' (>1000).

The most distantly related organism to possess two dystrobrevin SBSs is the lamprey (*Petromyzon marinus*), which has sequences orthologous to exon 12 and exon 13 in its genome. Those animals more divergent than the lamprey (amphioxus, sea squirts and all other invertebrates) have the single E14 SBS [1], while all those more closely related to mammals (all vertebrates including cartilaginous fish) have multiple paralogous dystrobrevins [1], each with two or three SBSs. We used primers based on the *P. marinus *sequence to amplify dystrobrevin transcripts from whole-body RNA of the closely related species *Lampetra planeri*. This gave a fairly equal mixture of two isoforms, corresponding exactly to the mammalian 'a' and 'c' *fine *isoforms ([GenBank: FJ535567] and [GenBank: FJ535566], respectively), showing that this pattern of alternative splicing is ancient (Figure 4B). By comparison, our work on zebrafish α-dystrobrevin shows that most transcripts contain E12 but not E13, and that inclusion of exon 9 is rare [1]. Splicing of exon 10 to exon 14 (the 'a' form) was not observed in zebrafish.

### The novel SBS binds syntrophin

In order to assess whether exon 11b is likely to be able to bind syntrophins, we compiled an alignment of representative known and suspected SBSs from vertebrate dystrobrevins (Figure 4A), and generated sequence "logos" from a range of tetrapod representatives of each category of SBS using WebLogo [48]. Dystrobrevin exon 14, which encodes SBS2, is extremely highly conserved across all species, with a core SBS (an almost invariant 15-residue LDEEHRLIARYAARL motif) set within an extended conserved consensus. Exons 11b from α-dystrobrevins (encoding SBS1') and exons 13 from α-, β- and γ-dystrobrevins (encoding SBS1) each form their own conserved clades with their own characteristic variants of the SBS motif. Thus within the generalised consensus of LA+EHxLIxxYVxxL, β-dystrobrevin SBS1 have LADEHALIASYVARL, α-dystrobrevin SBS1 have LADEHVLIGLYVNML, and α-dystrobrevin SBS1' have VAEEHSLIKLYVNQL. Within each sequence family, this motif is virtually invariant (Figure 4A). Phylogenetic analysis (data not shown) places the amino acid sequence of SBS1' firmly within the dystrobrevin SBSs, and specifically suggests that it arose through a serial duplication of α-dystrobrevin SBS1 rather than by a parallel independent duplication of SBS2. This is confirmed by phylogenetic analysis of the encoding exonic DNA sequences. A striking feature of SBS1', when compared with all others, is the invariant presence of the lysine residue at position 9. This position contains a small amino acid (either alanine or glycine) in all other dystrobrevin SBSs, suggesting that the introduction of the large, positively charged lysine might have a substantial impact on binding specificity.

To test whether the newly identified SBS1' encoded by exon 11b can actually bind syntrophin, we generated a series of α-dystrobrevin yeast two-hybrid (Y2H) constructs corresponding to each of the human one- or two-SBS isoforms. We also generated mutants bearing leucine-to-proline substitutions at position 7 in one or both SBSs (arrow in Figure 4A; see Additional file 2 for details). These were tested for their ability to interact with full-length rat β1-syntrophin (encoded by a clone previously identified from a Y2H screen using DRP2 as a bait [49]). These experiments showed that, at least in the context of a yeast nucleus, β1-syntrophin interacts robustly with all three α-dystrobrevin SBSs, and that the leucine-to-proline mutations ablate this interaction in each case (Figure 4C). Critically, the b-isoform still interacts when a mutation known to abrogate binding via SBS2 is introduced (compare a^w ^and a^m ^with b^ww ^and b^wm^), and this interaction is in turn abolished when an analogous mutation is introduced into SBS1' (compare b^wm ^and b^mm^). Thus, in the context of the Y2H system, exon 11b encodes a functional SBS. Also, even in this relatively non-quantitative assay, constructs with two intact SBSs resulted in substantially higher growth than those with one (compare b^ww ^and c^ww ^with other constructs), suggestive of the simultaneous binding of multiple syntrophin molecules per dystrobrevin molecule, as expected [37].

## Discussion

The most novel aspect of this study is the description of a previously unrecognised third SBS (SBS1') encoded by the α-dystrobrevin gene of most tetrapods. We assume that the late recognition of a major novel coding exon in a gene which has been intensely studied since its discovery thirteen years ago [6,8] is due to the predominance of the mouse as the model organism and the ablation of SBS1'-encoding exon 11b in the murid lineage. An interesting implication of the existence of two alternative two-SBS α-dystrobrevin isoforms ('b' and 'c', i.e. containing exons 10-11b-14 and 10-12-13-14) is that the regulation of α-dystrobrevin SBS composition seems to be about more than mere stoichiometry. Indeed this suggests that there is substantial specificity of syntrophin-SBS interaction such that not only the stoichiometry, but also the *flavour *of the DGC can be manipulated; this is consistent with a previous study of interactions between SBSs and the five vertebrate syntrophins, which shows a certain degree of binding specificity [39]. The typical vertebrate has eleven SBSs, which fall into roughly seven distinct sequence classes (Roberts, unpublished observations); within each sequence class, idiosyncrasies have been strongly maintained over hundreds of millions of years (see sequence logos at bottom of Figure 4A), suggesting that some sort of functional specificity is to be expected. Only trace amounts of a three-SBS 'd' isoform of α-dystrobrevin, which would increase the potential stoichiometry of syntrophins up to five per DGC, were observed in the tissues tested. It has to be said that although apparently stringently regulated, the consequences of variable SBS and syntrophin composition for DGC function remain far from clear.

A second major aspect of this study is the conclusion that brain-specific transcription commencing in intron 7 of the α-dystrobrevin gene (using exon 7b), also first described thirteen years ago [8], probably occurs in most tetrapods, but not in mice. This transcription represents a substantial proportion of all α-dystrobrevin transcription in human brain, and should give rise to the severely N-terminally truncated α-dystrobrevin-4 and -5 isoforms [8]. Translation is likely to start at the methionine 319 codon in exon 8, a residue which is conserved in most α-dystrobrevins. These brain-specific proteins would range in size from 22 kDa for the shortest α-dystrobrevin-5 to 48 kDa for the longest α-dystrobrevin-4 isoform. They would be able to bind syntrophins and dystrophins, but would lack the EF hands and ZZ domain. It is interesting that since the first description of α-dystrobrevin-4 and -5 [8], and despite their frequent depiction in diagrams in the literature, these isoforms have barely been studied. We attribute this omission, like our ignorance of exon 11b, to the predominance of mouse as a model organism.

The recent availability of several rodent genome sequences has allowed us to refine the period during which the simplification of the α-dystrobrevin gene occurred. Both exon 11b and exon 7b are intact in *S. tridecemlineatus*, *C. porcellus*, and *D. ordii *but clearly disrupted in *M. musculus *and *R. norvegicus*. Dating of the flanking divergence events (*M. musculus*/*R. norvegicus *and Muroidia/Geomyoidia) constrains these events to ~20-60 million years ago (Mya) [50]. Further constraint is possible in the case of exon 7b, which is also found to be disrupted in the deer mouse *Peromyscus maniculatus *(exon 11b is missing from this animal's sequence trace archive). The finding of this mutation in *P. maniculatus*, which is in family Cricetidae, is the first indication that some non-murid members of superfamily Muroidea (including hamsters and voles) are also highly likely to have a simplified α-dystrobrevin repertoire; it also narrows the mutation events to ~30-60 Mya [50]. We are currently unable to resolve the order of the two disruptions, and it is possible that they occurred in quick succession in common response to a single ancestral change in brain-specific α-dystrobrevin requirements.

## Conclusion

A major consequence of our findings is the supposition that of all mammals, mouse (and its relatives the rat and hamster) is likely to be a particularly poor model in which to study the function of both α-dystrobrevin and the DGC in the nervous system. Although much emphasis has been rightly placed on the effects of DGC disruption on skeletal and cardiac muscle, many DMD patients have substantial neurological problems, including learning difficulties [12,13] (30% of DMD patients have an IQ<70, and in 6%, IQ<50; this correlates strongly with mutation site, implicating short dystrophin isoforms in cognitive function [13]), congenital stationary night-blindness [51], defective colour vision [52] and a suggestion of personality disorders [53-55]. In addition, α-dystrobrevin binds dysbindin (also known as DTNBP1) [56], a protein whose gene has been associated with schizophrenia and other psychiatric disorders [57,58]. The brain is the major site of α-dystrobrevin expression, α-dystrobrevin is the predominant dystrophin partner in the brain, and α-dystrobrevin *coarse *isoforms have been shown to be expressed in a stringently cell-type-specific manner in the brain [59,60]; the absence from mouse of >50% of the brain α-dystrobrevin isoforms means that the mouse brain DGCs are highly atypical in composition.

## Abbreviations

cDNA: complementary deoxyribonucleic acid; CNS: central nervous system; DGC: dystrophin glycoprotein complex; DMD: Duchenne muscular dystrophy; DRP2: dystrophin-related protein 2; EST: expressed sequence tag; MaLR: mammalian apparent LTR-retrotransposon; Mya: million years ago; NMJ: neuromuscular junction; nNOS: neuronal nitric oxide synthase; PDZ: postsynaptic density protein 95/discs large/zonula occludens-1 (domain); PH: pleckstrin homology (domain); RT-PCR: (reverse transcript) polymerase chain reaction; SBS: syntrophin-binding site; SU: syntrophin unique (domain); TFBS: transcription factor binding site.

## Authors' contributions

SVB performed all human and mouse quantitative experiments and helped write the manuscript. PC generated wild-type and mutant yeast two-hybrid constructs. ST and HJ performed lamprey and preliminary human analysis. RGR performed bioinformatic analyses, conceived the project and wrote the paper.

## Supplementary Material

Additional file 1 **Tables S1-S5**. Quantitative and Non-Quantitative RT-PCR Analysis. Click here for file

Additional file 2 **Figure S1**. Generation of α-Dystrobrevin SBS Constructs for Yeast Two-Hybrid. Click here for file

Additional file 3 **Figure S2**. Conservation of Exon 7 and α-Dystrobrevin-4 and -5 First Exon (Exon 7b). Click here for file
