# Supplementary material for: Profound human/mouse differences in alpha-dystrobrevin isoforms: a novel syntrophin-binding site and promoter missing in mouse and rat
Source: BMC Biol. 2009 Dec 4;7:85. doi: 10.1186/1741-7007-7-85 (PMC2796648; doi:10.1186/1741-7007-7-85)
Supplement: Additional file 1 — Tables S1-S5. Quantitative and Non-Quantitative RT-PCR Analysis. [file 1741-7007-7-85-S1.PDF]

Supplementary Tables 1-5: Quantitative and Non-Quantitative RT-PCR Analysis

Initially, we used nested RT-PCR reactions spanning the SBS-encoding region. The products of this reaction were dominated by sequences in which exon 10 is joined directly to exon 14; this is entirely expected, not only because this is the most prevalent species in dbEST, but also because competing PCR reactions of different template size usually favour the smaller product. We therefore decided to do two things to reduce sources of bias: a) to use a large first-round PCR product, thereby reducing the proportional length differential between isoforms; b) to use isoform-specific primers which traverse exon boundaries (10-11b, 10-12, 10-13, 10-14, 11b-12, 11b-13, 11b-14, 12-13, 12-14, 13-14). This allowed us to establish the repertoire of transcripts from among all possible exon combinations.

We then cloned isoform-specific PCR products for each of these isoforms, starting across the E8-E9 or E8-E10 boundary for + and – isoforms, respectively (in humans; in mouse, we did not aim to distinguish + and – isoforms), and ending at isoform-specific boundaries, namely the E10-E14, E11b-E14, and E10-E12 boundaries for isoforms a, b, and c, respectively (other products were also cloned, including the three-SBS isoform E10-E11b-E12-E13-E14, "d" but as only trace amounts of these were seen in the later quantitative analysis, these have been omitted). The products were cloned into pCR4 (Invitrogen) and serially diluted to form standard series for quantitative analysis of endogenous transcripts. The specificity of each reaction was tested by comparing the performance of primers against both their cognate standard series and their most closely related standard. In cases where specificity failed to be maintained over the whole dynamic range of the standard series (presumably due to cross-hybridization of primers, as the exon-boundary primers would often share 50% of their length), new primers were designed and tested.

Semi- and non-quantitative RT-PCR: Single-strand cDNA was synthesized from equal amounts of total RNA using gene-specific primers (HADybE15RO, HADybI18R and HADybE19R, or their mouse equivalents; Tables S1, S3) and M-MLV Reverse Transcriptase (Applied Biosystems). 2 µl cDNA was used for the first round of nested PCR using "coarse" isoform-defining primer pairs (HADybE7F or HADybE7bFO, with HADybE15RO, HADybI18R or HADybE19R, or their mouse equivalents; Tables S1, S3). Each 50-µl PCR reaction contained 20 pmol of each primer, 1.5 units of Platinum *Taq* DNA Polymerase High Fidelity (Invitrogen), High Fidelity Buffer (final concentration 60 mM Tris-SO_4, pH8.9, 18 mM $(NH_4)_2SO_4$), 0.2 mM $MgSO_4$ and 0.1 mM dNTPs. All reactions were incubated for 10 min at 95°C and then cycled (20 times for first-round, 20 times for second-round PCR) through 45 s 95°C, 45 s 58°C and 45 s 72°C, followed by a final incubation of 10 min at 72°C.

Quantitative RT-PCR: Every 20-µl reaction contained 2 µl template, 10 µl 2x Precision MasterMix premixed with SYBR green (PrimerDesign), 5 pmol of each primer (Supplementary Tables 2 and 4) and 7 µl H_2O. Q-PCR was performed using 96-well half-skirted PCR plates (Anachem) with seals (ABsolute™ QPCR Seal, ABGene) on an ABI Prism 7000® Sequence Detection System.

Supplementary Tables 1-4: primer combinations used

Supp Table 1: Outer primers for human QRT-PCR (by coarse isoform)

Isoform	Forward	Reverse
**ADyb-1**	HADybE7F	HADybE19R
**ADyb-2**	HADybE7F	HADybI18R
**ADyb-3**	HADybE6F	HADybE11RO
**ADyb-4**	HADybE7bFO	HADybE19R
**ADyb-5**	HADybE7bFO	HADybI18R

Supp Table 3: Outer primers for mouse QRT-PCR (by coarse isoform)

Isoform	Forward	Reverse
**ADyb-1**	MADybE7FO	MADybE19R
**ADyb-2**	MADybE7FO	HADybI18R
**ADyb-3**	MADybE7FO	MADybE11RO
**ADyb-4**	MADybE7bFI	MADybE19R
**ADyb-5**	MADybE7bFI	HADybI18R

Supp Table 2: Inner primers for human QRT-PCR (by isoform)

Isoform	Forward	Reverse
**1/2/4/5a**	HADybE9-F/ HADybE9+F	HADybE10-E12R
**1/2/4/5b**	HADybE9-F/ HADybE9+F	HADybE10-E14R
**1/2/4/5c**	HADybE10F	HADybE11b-E14R (2)
**3**	HADybE7F	HADybE11RI

Supp Table 4: Inner primers for mouse QRT-PCR (by isoform)

Isoform	Forward	Reverse
**1/2/4/5a**	MADybE7FI/HADybE8FI	MADybE10-E12R
**1/2/4/5c**	MADybE7FI/HADybE8FI	MADybE10-E14R
**3**	MADybE7FI	HADybE11RI

Supplementary Table 5: Primer Sequences

**Human**			**Mouse**			**Lamprey**		
**Name**	**Sequence**	**Usage**	**Name**	**Sequence**	**Usage**	**Name**	**Sequence**	**Usag[illegible]**
HADybE6F	CCCGCAGTGTCTGGTCTGGTTG	1st	MADybE7FO	GATGGGATTTCGATACCGATG	1st	LDybE7FO2	CTCGTTCTGCCACAGCGAGAG	1st
HADybE7F	CTGCTTCTGGAGGGGACATGCC	1st, 2nd	MADybE7FI	CTGCTTCTGGAGGGGCCATGCA	2nd, Q	LDybE7FI2	CAGCAGTGCCACAGCTACCAG	2nd
HADybE7bFO	GAACCCAGTGTAGCTTCCTGAG	1st	MADybE7bFO	TTGCCTGCCATTCTTTATCC	1st			
HADybE7bFI	GAATAGCAAGGACCTTCTGG	2nd, Q	MADybE7bFI	GTTTAGTGTGACTTCCTGAG	2nd			
HADybE8FO	GGAAATCACCTGCTAAGAAGCTG	1st						
HADybE8FI	GCCGTGAACCTTTGCACCCC	2nd	MADybE8FI	use HumADybE8FI	Q			
HADybE9-F	GCTCACATCGTGCCTCCCAG	2nd, Q						
HADybE9+F	GTTGATACTTGGCCTCCCAG	2nd, Q						
HADybE10F	GTTCCCTCCTCAGGAAGTCC	2nd, Q						
			MADybE10RO	GATGAAAGGACTTCCTGAGG	RT, 1st			
			MADybE10RI	ATGCTGGTTACAGGTCTCGG	2nd			
HADybE11RI	ATCCATCTAGACGCATCCACC	2nd, Q	MADybE11RI	Use HumADybE11RI	2nd, Q			
HADybE11RO	CTGGAGGAACCAGAACAGCTC	RT, 1st	MADybE11RO	GGAAGCAGAAAAGCTCAAGGGG	RT, 1st			
HADybE11bR	CGTGCACCGTGATCAAGCTG	2nd, Q						
HADybE10-E12R	GGGAGGAGAGCTCCTGGTAA	2nd, Q	MADybE10-E12R	GGGAGGAGAGCTCCTGGTGA	2nd, Q			
HADybE10-E13R	GTACTGTATCCTCCTGGTAA	2nd						
HADybE10-E14R	CTCAAGCATGCTCCTGGTAA	2nd, Q	MADybE10-E14R	CTCAAGCATACTCCTGGTGA	2nd, Q			
HADybE11b-E12R	GGGAGGAGAGCGTGCACCGT	2nd, (Q)						
HADybE11b-E13R	GTACTGTATCCGTGCACCGT	2nd, (Q)						
HADybE11b-E14R	CTCAAGCATGCGTGCACCGT	2nd						
HADybE11b-E14R2	GAACTCTCAAGCATGCGTGC	2nd, Q						
HADybE12-E13R	GTACTGTATCCCTTTGCCCT	2nd						
HADybE12-E14R	CTCAAGCATGCCTTTGCCCT	2nd						
HADybE13-E14R	CTCAAGCATGCATGAGGGGT	2nd						
HADybE15RI	GAAAGAGATGTCAGGAGCAC	2nd				LDybE15RI	GTGTCGGAGTTGAACGCCAAC	2nd
HADybE15RO	CTAGCTCAGCAATCAGCTGCC	RT, 1st				LDybE15RO	GATGAGCTGCCGCTGCTGCTTG	RT, 1[illegible]
HADybI18R	GACCTGCAGTAGGGGACATAAC	RT, 1st	MADybI18R	Use HumADybI18R	RT, 1st			
HADybE19R	GGGAATTGGCCTGCTGATGGTG	RT, 1st	MADybE19R	GGGATCGGCCTGCTGATGGTG	RT, 1st			
