# Supplementary material for: Profound human/mouse differences in alpha-dystrobrevin isoforms: a novel syntrophin-binding site and promoter missing in mouse and rat
Source: BMC Biol. 2009 Dec 4;7:85. doi: 10.1186/1741-7007-7-85 (PMC2796648; doi:10.1186/1741-7007-7-85)
Supplement: Additional file 2 — Figure S1. Generation of α-Dystrobrevin SBS Constructs for Yeast Two-Hybrid. [file 1741-7007-7-85-S2.PDF]

Supplementary Figure 1: Generation of α-Dystrobrevin SBS Constructs for Yeast Two-Hybrid

Sequences of α-dystrobrevin SBS constructs used in Y2H experiment to assess interaction with β1-syntrophin (red indicates sequences which differ from wild-type human α-dystrobrevin; green indicates expected intact SBSs):

	Exon 10	Exon 11b	Exon 12	Exon 13	Exon 14	Exon 15
a^{w}	VPSSGSPFITRS	------------------------------	--------------------------	-------------------------------	MLESSNRLDEEHRLIARYAARLAAESST	SQPPQQRS*
a^{m}	VPSSGSPFITRS	------------------------------	--------------------------	-------------------------------	MLESSNRLDEEHRPIARYAARLAAESST	SQPPQQRS*
b^{ww}	VPSSGSPFITRR	LPEGISASSPVAEEHSLIKLYVNQLDHGAR	--------------------------	-------------------------------	MLESSNRLDEEHRLIARYAARLAAESST	SQPPQQRS*
b^{mw}	VPSSGSPFITRR	LPEGISASSPVAEEHSPIKLYVNQLDHGAR	--------------------------	-------------------------------	MLESSNRLDEEHRLIARYAARLAAESST	SQPPQQRS*
b^{wm}	VPSSGSPFITRR	LPEGISASSPVAEEHSLIKLYVNQLDHGAR	--------------------------	-------------------------------	MLESSNRLDEEHRPIARYAARLAAESST	SQPPQQRS*
b^{mm}	VPSSGSPFITRR	LPEGISASSPVAEEHSPIKLYVNQLDHGAR	--------------------------	-------------------------------	MLESSNRLDEEHRPIARYAARLAAESST	SQPPQQRS*
c^{ww}	VPSSGSPFITRS	------------------------------	SPPKDSEVEQNKLLARAAPAFLKGKG	IQYSLNVADRLADEHVLIGLYVNMLRNNPSC	MLESSNRLDEEHRLIARYAARLAAESST	SQPPQQRS*
c^{mw}	VPSSGSPFITRS	------------------------------	SPPKDSEVEQNKLLARAAPAFLKGKG	IQYSLNVADRLADEHVPIGLYVNMLRNNPSC	MLESSNRLDEEHRLIARYAARLAAESST	SQPPQQRS*
c^{wm}	VPSSGSPFITRS	------------------------------	SPPKDSEVEQNKLLARAAPAFLKGKG	IQYSLNVADRLADEHVLIGLYVNMLRNNPSC	MLESSNRLDEEHRPIARYAARLAAESST	SQPPQQRS*
c^{mm}	VPSSGSPFITRS	------------------------------	SPPKDSEVEQNKLLARAAPAFLKGKG	IQYSLNVADRLADEHVPIGLYVNMLRNNPSC	MLESSNRLDEEHRPIARYAARLAAESST	SQPPQQRS*
d^{www}	VPSSGSPFITRR	LPEGISASSPVAEEHSLIKLYVNQLDHGAR	SPPKDSEVEQNKLLARAAPAFLKGKG	IQYSLNVADRLADEHVLIGLYVNMLRNNPSC	MLESSNRLDEEHRLIARYAARLAAESST	SQPPQQRS*

Construct "a" corresponds to nucleotides 1124-1267 of U46744, "b" 244-477 of FJ535565 (431-664 of AK295789), "c" 1322-1636 of U46745 and "d" 244-648 of FJ535564 (443-847 of AK295732).

Primers used to amplify and mutate α-dystrobrevin SBS constructs (red indicates sequences which differ from wild-type human α-dystrobrevin; underline indicates restriction sites used) are as follows:

ADybSBSF: ccatggttccctcctcaggaagtcc

ADybSBSR: ggatcctcaacttctctgctgaggtggctgagacgtcgaggac

SBS11bmutF: ggttacctgagggaataagtgcatccagccctgtggctgaagagcattccccata

SBS13mutF: gctagctgatgaacatgttcccatc

SBS14mutR: gacgtcgaggactctgctgccagccttgccgcatacctggcaattggcc
