# Supplementary material for: Profound human/mouse differences in alpha-dystrobrevin isoforms: a novel syntrophin-binding site and promoter missing in mouse and rat
Source: BMC Biol. 2009 Dec 4;7:85. doi: 10.1186/1741-7007-7-85 (PMC2796648; doi:10.1186/1741-7007-7-85)
Supplement: Additional file 3 — Figure S2. Conservation of Exon 7 and α-Dystrobrevin-4 and -5 First Exon (Exon 7b). [file 1741-7007-7-85-S3.PDF]

Supplementary Figure 2: Conservation of Exon 7 and α-Dystrobrevin-4 and -5 First Exon (Exon 7b)

Canonical exon 7 is highly conserved in all mammals and, indeed, all animals (it encodes the entire ZZ domain). There is a surprising degree of conservation at the third (wobble) position of codons, which may be due to overlapping functional constraint as a promoter for α-dystrobrevin-4 and -5 transcripts (several highly conserved bases are uniquely mutated in mouse, rat and deer mouse).

The first exon of α-dystrobrevin-4 and -5 isoforms (exon 7b) is conserved in most mammals, including its donor splice site. There is evidence of use in humans (50 ESTs), chimpanzee (3 ESTs), cow (2 ESTs) and macaque (1 EST). It is used in opossum (1EST), but a different donor splice site is used as the canonical eutherian one is mutated. In the murid species (mouse and rat; also deer mouse), there is no EST evidence for usage, we could not amplify it from mouse brain RNA, the donor splice site has a 1-bp insertion mutation, and the exon has experienced a murid-specific insertion of a MaLR family LTR sequence.

EST/CAGE evidence for transcriptional start sites (TSSs) is shown by vertical bars. Black bars represent 5' ends of ESTs; only ESTs which contained the 3' end of the first exon and which spliced clearly onto canonical exon 8 were considered. The smallest bars represent one EST starting at that base (the largest bar represents 14 ESTs). Red bars represent 5'cap-trapped CAGE tags, which are strongly indicative of genuine RNA polII initiation events rather than potentially premature reverse transcriptase termination events. Bars above the sequence are human; those below are non-human, with species indicated. No relevant mouse or rat ESTs were observed.

```
Human       ccaaat--caactatcat-ctgaaatc---gtggtgcagagaagaaaggtgcaattcctgtaaggct-gtatttttccttcttctggttcatacttttgt
Macaque     ccaaat--caactatcat-ctgaaatc---gtggtgcagagaagaaaggtacaattcctgtaaggct-gtatttttccttcttctggttcatacttttgt
Mouse       caaaata--gatcatggt-ctgaaatcagcagagcacagagctgaaaaattgaattctatgaaagct-gtggttttctttagtttagttcacaattctat
Rat         cagaat--------tggt-ctgaaatcagcagggcacacagctgaagaacagaattccccgagagag-gtgtttttctt--gtctagttcacactcctgt
DeerMouse   caaagtt--gatcatggt-ctgaaatcaccacagggcacagttgaaaaaaatagaattccttgtagttgtgtttttctttagtttagttcacacttgtcc
KangRat     caaaatt--atctatggt-ttgaatttcatagaacgtagcagaaagctctagttctttcta--ggct-gcctttttcttcttctggctcatactttgtt
GuineaPig   taatatt--aactatcgt-ctgaagac---atgttatacagcagaa-aatgtgatttcatcggaact-gggtttttccttattctggttcataccgtcat
Rabbit      caaaacc--gactatcac-ctgaaatc---atggccctcagtggaaaagtgctttgctatt-gagct-gtgtttttccttattctggttcatacttttgc
Dog         caaaatt--gactatcat-ctgaaatcatggcaccatggcacccagcagaaaagctcctgatagact-gtaggtttccttattctggctcatgcttgtat
Cow         ccaag------ctatcat-cagaagtc---ttgttattcagcagaaaaatgcaattcttgttgggct-gtatttttcattcttctgactcattctcttgt
Armadillo   caagatg--gattatcac-ctgaaacc---atagcctatggcaggagaatgcaattcctgttaggtt-atgt-ttttttcgttct-gttaattctttttt
Elephant    caagact--gaccatcat-ctaaagtc---atggcacacagcagaaaaatccaattc-tattaagct-atatgtttccttattat-gttcattctttttt
Opossum     ctgaatgtcaaatagaatgctaaagattagg-gc-agagagtggaagaatttagtgtcttttagg------agtttatctttaatattatccattttga
Platypus    ccaaagg-cactctggatgctgaaaatggtttgt-gcccaatggaaggattcattgccgaggcgg------tgttttatttttaatggtgttc-tgttgt
Chicken     agtcaacacctgatgtcacagattcct---------aggaaaaaaaaaaaagtgattcactcttccat-tcagagtttttcacatcagcttttccttgggt
Lizard      tggcatttaaatttcagcccataccaaaagagcatttcatactgaaaatcaacagatacaaagggaa-gctcttctaatggatttggatttaatgctgca
Frog        ------------------------tagggatcattgatatattgatttatttatagaataaggcttg-tggcttaggagctgtggatacaaaagctttgt
ZebFish     ----------------------------------------------------------------gga-gcattattatatagtgttgttactatacaacc

Human       tttc------ttg-gtttttcttcacttactt-cccccttagTCTTCCATCCGGTTGAGTGTTCCTACTGCCACAGTGAGAGTATGATGGGATTTCGCTA
Macaque     tttc------ttg-ttttttcttcacttactt-cccccttagTCTTCCATCCGGTGGAGTGTTCCTACTGCCACAGTGAGAGTATGATGGGATTTCGCTA
Mouse       ggct----ttttc----ccccctcatttacgt-cctccctagTCTTCCATCCAGTTGAGTGTTCCTACTGTCACAGTGAGAGCATGATGGGATTTCGATA
Rat         tttt----tttttttttcttcctcatttactt-cctccctagTCTTCCATCCGGTTGAGTGTTCCTACTGTCACAGTGAGAGCATGATGGGATTCCGATA
DeerMouse   tgat-----------tttttcctcatttactttccttcctagTCTTCCATCCAGTTGAGTGTTCCTACTGTCACAGTGAGAGCATGATGGGATTTCGCTA
KangRat     ctct--------tgttttgttttgttttttcc-attttctagTCTTCCACCCAGTTGAGTGTTCTTACTGCCACAGTGAAAGTATGATGGGATTTCGCTA
GuineaPig   gt--------tcctgtttttcttcgtttactc-cccccttagTCTTCCATCCGGTTGAGTGTTCCTACTGCCACAGCGAGAGTATGATGGGATTTCGCTA
Rabbit      tttc----ttgtt---ttgtctccatgtactc-tccccttagTCTTCCATCCGGTGGAGTGTTCCTACTGCCACAGTGAGAGTATGATGGGATTTCGCTA
Dog         ttttctctttttttttttttctccatttcatt-cccccttagTCTTCCATCCGGTTGAATGTTCTTACTGCCACAGTGAGAGTATGATGGGATTCCGCTA
Cow         ttcc------ttgtctttttcttcgtttgctt-gcccgtcagTCTTCCACCCGGTGGAGTGTTCATACTGCCACAGTGAGAGTATGTTGGGCTTTCGCTA
Armadillo   t-----------------cccttcatttactt-cccccttagTCTTCCATCCGGTTGAGTGTTCCTACTGCCACAGTGAGAGTATGATGGGATTTCGCTA
Elephant    taaa-----tttc----cttcttcatttattt-ctccc-tagTCTTCCATCCAGTTGAGTGTTCCTACTGCCACAGTGAGAGTATGATGGGATTTCGCTA
Opossum     t---------ttttttaaattttaattcattt-tcccctcagTCTTCCACCCCGTTGAGTGTTCCTACTGCCACAGTGAGAGCATGATGGGATTTCGCTA
Platypus    t---------tttgttta--tttcattcatct-ctctcttagTCTTCCACCCTGTTGAGTGTTCCTACTGCCATAGTGAGAGCATGATGGGATTTCGCTA
Chicken     tcctctgcttggggttaaagcttggcgtctct-ctcccacagTCTTCCATCCTGTTGAGTGTTCCTACTGCCACAGCGAGAGCATGATGGGGTTCCGCTA
Lizard      ttgattgttttgtttttctgttaaatttattt-cccttgtagTCTTCCACCCTGTTGAGTGTTCCTACTGCCATAGCGAGAGCATGATGGGATTTCGCTA
Frog        atggactgatgaatgataatgtctgtattttt-gccccatagTCTTCCATCCTGTTGAGTGTTCCTATTGCCACAGTGAGAGCATGATGGGTTTTCGTTA
ZebFish     agattgtaaagtgtaccaattttatctggtct-cctctgtagTTTTTCATCCAGTGGAGTGTTCATACTGCCACAGCCAGAGTATGATGGGATTTCGCTA
```

Canonical Exon 7

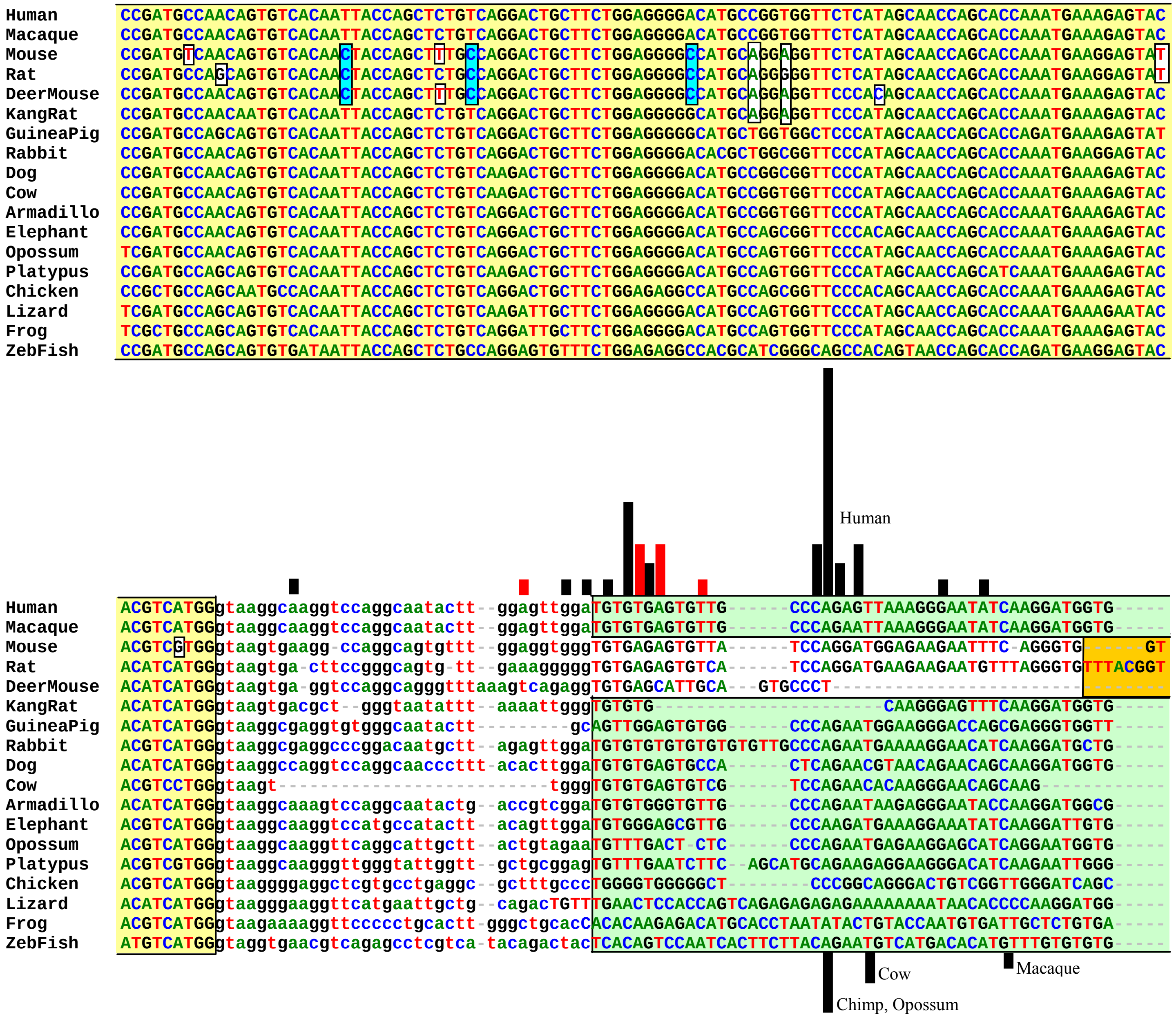

Within exon 7, cyan boxes indicate changes seen in mouse, rat and deer mouse but in no other mammalian sequences. White boxes indicate other murid differences.

Exon 7b

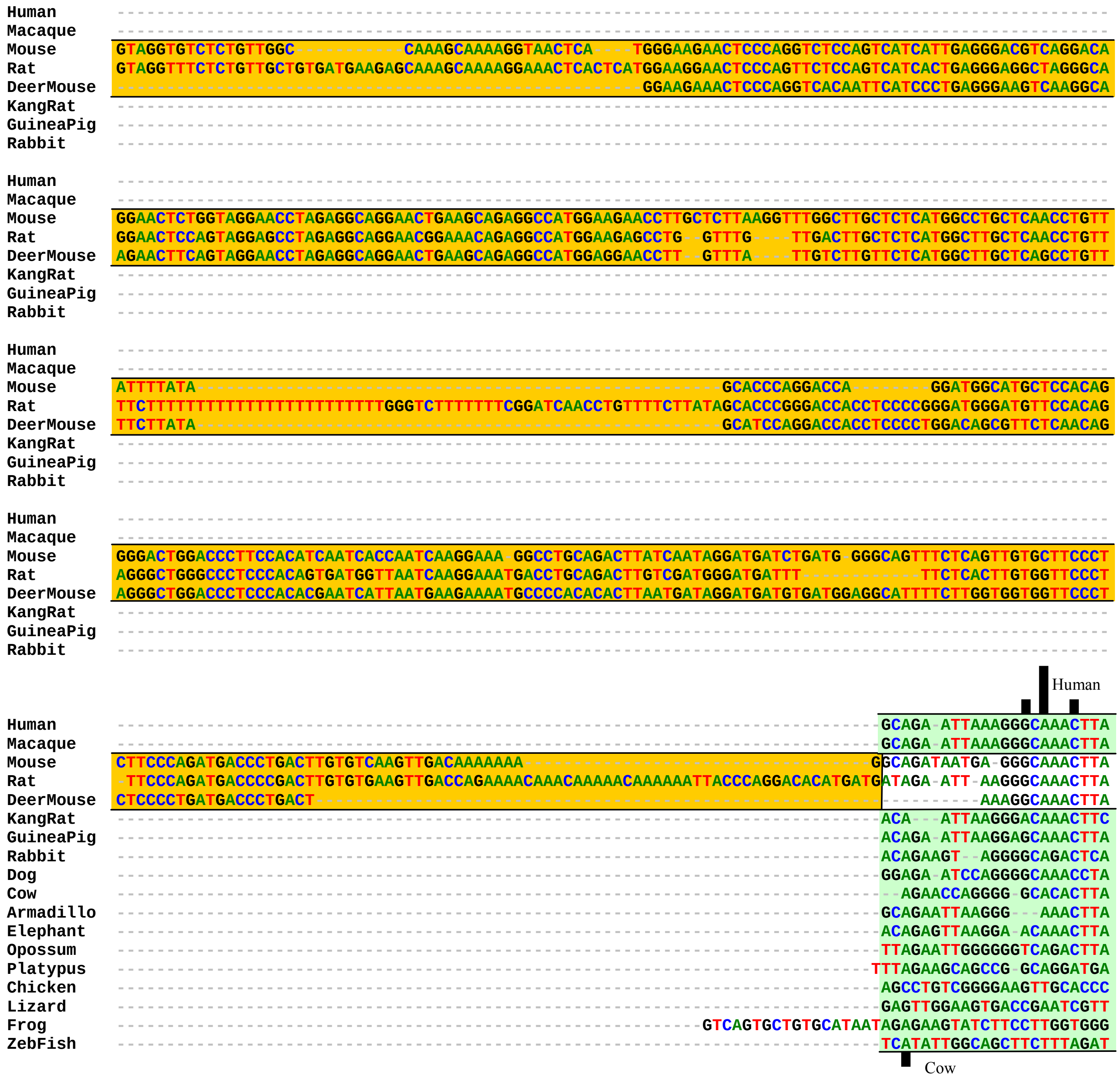

Murid-specific MaLR LTR Insertion

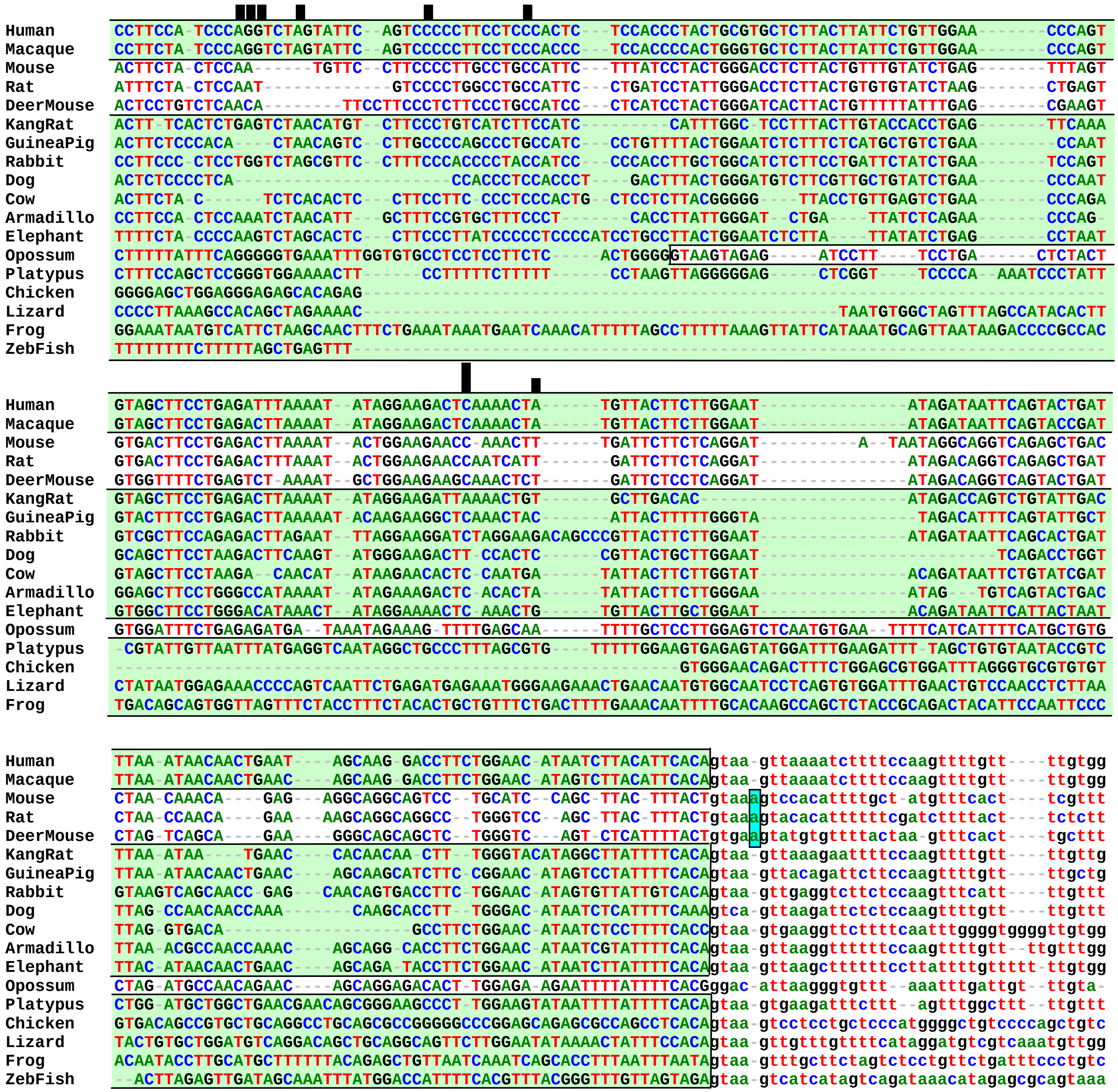

Opossum donor site used in single *Trichosurus vulpecula* EST (EC290283).

Exon 7b

Exon 7b donor site used in all human, chimp, macaque and cow ESTs. Bears an insertion mutation in mouse, rat and deer mouse (cyan box) and is obliterated in opossum.
